# Epidemiology of Generalized Joint Laxity (Hypermobility) in Fourteen-Year-Old Children From the UK: A Population-Based Evaluation

**DOI:** 10.1002/art.30435

**Published:** 2011-09

**Authors:** Jacqui Clinch, Kevin Deere, Adrian Sayers, Shea Palmer, Chris Riddoch, Jonathan H Tobias, Emma M Clark

**Affiliations:** 1 Bristol Royal Hospital for ChildrenBristol, UK; 2 University of Bristol and Southmead HospitalBristol, UK; 3 University of the West of EnglandBristol, UK; 4 University of BathBath, UK

## Abstract

**Objective:**

Although diagnostic criteria for generalized ligamentous laxity (hypermobility) in children are widely used, their validity may be limited, due to the lack of robust descriptive epidemiologic data on this condition. The present study was undertaken to describe the point prevalence and pattern of hypermobility in 14-year-old children from a population-based cohort.

**Methods:**

We performed a cross-sectional analysis using the Avon Longitudinal Study of Parents and Children, a large population-based birth cohort. Hypermobility among children in the cohort (mean age 13.8 years) was measured using the Beighton scoring system. Objective measures of physical activity were ascertained by accelerometry. Data on other variables, including puberty and socioeconomic status, were collected. Simple prevalence rates were calculated. Chi-square tests and logistic regression analyses were used to assess associations of specific variables with hypermobility.

**Results:**

Among the 6,022 children evaluated, the prevalence of hypermobility (defined as a Beighton score of ≥4 [i.e., ≥4 joints affected]) in girls and boys age 13.8 years was 27.5% and 10.6%, respectively. Forty-five percent of girls and 29% of boys had hypermobile fingers. There was a suggestion of a positive association between hypermobility in girls and variables including physical activity, body mass index, and maternal education. No associations were seen in boys.

**Conclusion:**

We have shown that the prevalence of hypermobility in UK children is high, possibly suggesting that the Beighton score cutoff of ≥4 is too low or that this scoring is not appropriate for use in subjects whose musculoskeletal system is still developing. These results provide a platform to evaluate the relationships between the Beighton criteria and key clinical features (including pain), thereby testing the clinical validity of this scoring system in the pediatric population.

Joint hypermobility results from ligamentous laxity (1) and may occur in individuals with a primary genetic disorder affecting connective tissue matrix proteins (such as osteogenesis imperfecta or Marfan syndrome) or other syndromes, including trisomy 21, bony dysplasias, or velocardiofacial syndrome. In the majority of cases hypermobility exists as an isolated finding (referred to below as “generalized joint laxity”), but it may be associated with musculoskeletal symptoms such as pain and “clicking joints” in the absence of known genetic causes, in which case it is referred to as “hypermobility syndrome.”

The extent to which generalized joint laxity is associated with significant clinical sequelae, including joint pain, is unclear, since previous studies linking generalized joint laxity with joint pain in school children had limitations related to sample size, methods of assessing hypermobility, and methods of assessing pain. Despite this, data from studies of school-based populations suggest that the prevalence of pain among children with generalized joint laxity ranges from 30% (2) to 55% (3). An alternative view, namely, that generalized joint laxity as generally defined represents part of the normal population variance and that any association with joint pain is spurious (4), is also plausible. Current understanding of the prevalence and descriptive epidemiology of generalized joint laxity in childhood is limited, making it difficult to draw clear conclusions about causal pathways.

The reported prevalence of generalized joint laxity in children ages 6–15 years varies between 8.8% (5) and 64.6% (6). One explanation for the wide range of these prevalence estimates is that previous studies have been performed on selected populations (5–12). For example, some studies used preschool children ages 4–7 years (6), others used children ranging in age from 5 to 17 years and from a single school, with the report including no explanation of recruitment methods (5, 12), and sample sizes in previous studies were generally small, ranging from 364 children (9) to 2,432 children (5). All of these points reflect the fact that true population-based studies have not previously been undertaken.

Another explanation for the widely varying estimates of prevalence relates to differences in definitions. All of the above-mentioned studies used the method of examining and scoring for hypermobility developed by Beighton et al (13). The Beighton score was devised in South Africa and based on 1,083 Tswana Africans (adults and children), adapting a score previously described (in 1960) by Carter and Wilkinson (14). The Beighton score has subsequently been used internationally to define generalized joint laxity in all populations and all age groups. Most of the available prevalence studies used different cutoffs, ranging from ≥3 hypermobile joints to ≥6 hypermobile joints of 9 assessed (both thumbs, both little fingers, both elbows, both knees and the trunk) (Figure 1), and in some, only the dominant side was assessed. The most frequent choice of cutoff was ≥4 hypermobile joints.

**Figure 1:**
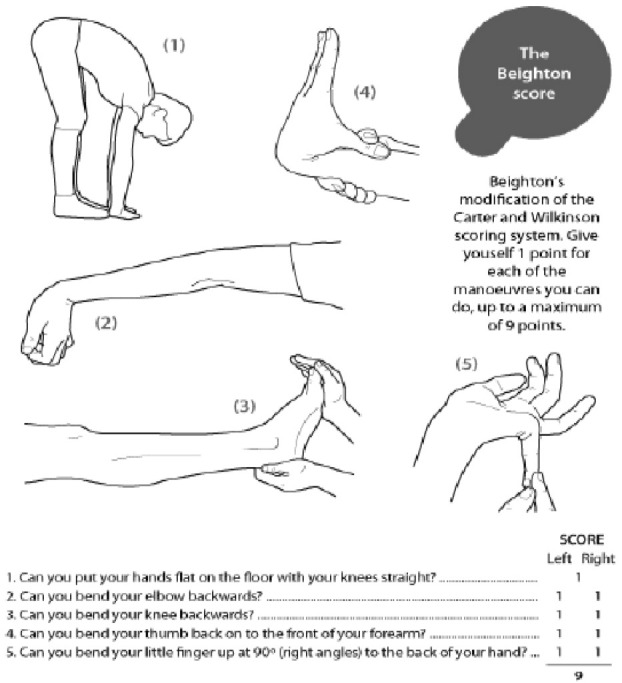
Calculation of the Beighton score. Reproduced, with permission, from Arthritis Research UK (http://www.arthritisresearchuk.org/arthritis_information/arthritis_types__symptoms/joint_hypermobility.aspx#non).

Although there is some published information about the descriptive epidemiology of generalized joint laxity, the studies were largely performed in selected groups, making it difficult to draw definitive broad conclusions. For example, generalized joint laxity is thought to be more common in girls compared to boys (5, 9, 15). There is also a suggestion that ethnic background can influence hypermobility (16, 17) and that generalized joint laxity is more common in ballet dancers (18), musicians (19), gymnasts (20), and swimmers (21). Contradictory results from some small studies have demonstrated greater degrees of joint laxity in either the dominant limb (22) or the nondominant limb (23). A lack of association with body weight has been reported consistently (8, 24, 25).

It is also widely believed that younger children are more flexible than adolescents (26), but there is very little literature to support this. For example, one rigorous population-based study from Sweden (15) investigated 1,845 children ages 9, 12, or 15 years from 48 geographically randomly selected schools and showed that at all ages, girls had a higher degree of generalized joint laxity as assessed by the modified Beighton criteria. However, joint laxity in boys decreased with increasing age, whereas girls had the highest degree of general joint laxity at the age of 15 years. Similarly, a study of high school basketball players (27) showed that after the onset of puberty, girls exhibited greater joint laxity than boys. Conversely, other studies have shown no decline in generalized joint laxity with age (28).

Therefore, to provide a basis for exploring relationships between generalized joint laxity and clinical sequelae, we aimed to define the prevalence and descriptive epidemiology of this condition. We performed a cross-sectional analysis of subjects in the Avon Longitudinal Study of Parents and Children (ALSPAC), based on Beighton scores obtained at the ALSPAC research clinic for 14-year-olds.

## SUBJECTS AND METHODS

### Study design and population

This was a cross-sectional analysis of a large population-based cohort, the ALSPAC. The ALSPAC (http://www.alspac.bris.ac.uk) is a geographically based UK cohort study for which pregnant women residing in Avon (southwest England) with an expected date of delivery between April 1, 1991 and December 31, 1992 were recruited (29). A total of 14,541 pregnant women were enrolled, with 14,062 children born. Of these births, 13,988 children were alive at age 12 months. The study is based on 6,022 children who attended the research clinic for 14-year-olds and had hypermobility data collected. Compared to the complete cohort, those included in this study of generalized joint laxity were more likely to have mothers educated to a university degree level or higher (17.1%, versus 9.4% of mothers of children not included in this analysis; *P* < 0.001). Ethics approval was obtained from the ALSPAC Law and Ethics Committee and the local research ethics committees. Parental consent and child's assent were obtained for all measurements.

### Measurement of generalized joint laxity

Generalized joint laxity was assessed by trained measurers in the research clinic for 14-year-olds, using the modified Beighton 9-point scoring system (13). Each joint was assessed separately (Figure 1). The fifth metacarpophalangeal joint was scored as hypermobile if it could be extended >90°, the thumb was scored as hypermobile if it could be opposed to the wrist, the elbows and knees were scored as hypermobile if they could be extended >10°, and the trunk was scored as hypermobile if both palms could be placed flat on the floor with the knees straight. Scores were recorded for the individual joints, and a total score (of a maximum of 9) was ascertained. A cutoff of ≥4 hypermobile joints was used to define generalized joint laxity, based on the cutoff most commonly cited in the literature (6–9). In addition, a more extreme phenotype was selected, with a cutoff of ≥6 hypermobile joints (reported to be the median number in children with any hypermobile joints [4]) to allow simple sensitivity-type analyses for confirming any associations found.

### Other measures

#### Anthropometric features

At the research clinic for 14-year-olds, height was measured to the last complete millimeter, using a Harpenden stadiometer. Weight was measured to the nearest 50 gm using a body fat analyzer (model TBF 305; Tanita). Body mass index (BMI) was calculated as kg/m^2^ and subjects were categorized as underweight (BMI <18.5), ideal weight (BMI 18.5–24.9), overweight (BMI 25–29.9), or obese (BMI >30) based on standard definitions.

#### Physical activity

Physical activity was measured objectively using an actigraph (model WAM 7164; MTI), for up to 7 days. For the purposes of this study, physical activity was categorized as ≥60 minutes versus <60 minutes of moderate and/or vigorous physical activity per day. This categorization has been previously described in detail (30). Briefly, a cut point of >3,600 counts per minute was used after calibration was performed in a subgroup of 260 children in whom these count frequencies were associated with oxygen consumptions of >4 metabolic equivalents (the ratio of the associated metabolic rate for the specific activity divided by the resting metabolic rate).

#### Socioeconomic status

The mother's highest education level was assessed at 32 weeks' gestation and was coded 1–5 where 1 = no formal qualifications or the lowest level of school educational qualification, 2 = vocational qualifications, 3 = O levels (generally gained at school by age 16 years), 4 = A levels (generally gained at school by age 18 years), and 5 = university degree. Other measures of the children's socioeconomic status, such as father's education, mother's and father's social class, and housing tenure where not used in this analysis as they yielded results similar to those obtained with the use of maternal education alone, as shown in a previous study on this cohort (31).

#### Others

Age was calculated from date of birth. Sex was ascertained from birth records. Hand dominance, or handedness, was recorded from data collected at research clinics the children attended at ages 7, 9, and 11 years as this is considered a stable trait. Puberty was assessed at age 13 using self-completion Tanner staging based on pubic hair distribution. The mother's, father's, and grandparents' race and ethnic group was recorded by the mother on self-reported questionnaires sent out at ∼32 weeks gestation, and based on this information, the child was categorized as white or nonwhite.

### Statistical analysis

All statistical analyses were carried out using Stata 11. Simple percentages were calculated for the point prevalence and pattern of generalized joint laxity. Chi-square tests were used to assess associations between binary variables and the presence or absence of generalized joint laxity. Logistic regression was used to analyze trends in associations between categorical variables and the presence or absence of generalized joint laxity. To evaluate the strength of associations, odds ratios (ORs) (with 95% confidence intervals [95% CIs]) for the presence or absence of generalized joint laxity according to each of the variables were calculated by logistic regression analysis. Multivariable logistic regression was used to evaluate independent associations. Interactions between sex and BMI were assessed by likelihood ratio test.

## RESULTS

The prevalence of generalized joint laxity, as defined using a Beighton score cutoff of ≥4 joints, in this population of 6,022 children (mean age 13.8 years) was 19.2%. The prevalence was higher among girls than among boys (27.5% versus 10.6%; *P* < 0.001). When a more rigorous cutoff was used (≥6 joints), the prevalence was 4.2% (7.0% in girls, 1.3% in boys; *P* < 0.001).

The distribution of hypermobile joints in the overall study population is shown in Table 1. The fingers were most likely to be hypermobile, followed by the thumbs. Knee, elbow, or trunk hypermobility was seen in ∼9% of the children. However, in girls, trunk hypermobility was more prevalent than elbow and knee hypermobility (15%, 13%, and 11%, respectively), while in boys, trunk hypermobility was unusual, with only 50 of 2,961 boys (1.7%) able to place both palms flat on the floor with the knees straight. Hypermobility of the thumb, knee, and elbow was found in 15%, 7%, and 4%, respectively, of the boys in the study. Among the children with hypermobility defined as ≥4 joints (n = 1,156), 85% had hypermobile fingers, 75% had hypermobile thumbs, and 29% had hypermobile knees (Table 2). Sex differences were seen, with 26% of the 842 girls with hypermobility exhibiting hypermobility of the trunk and 31% exhibiting hypermobility of the elbows, and only 4% and 21% of the 314 boys with hypermobility exhibiting hypermobility of the trunk and elbows, respectively.

**Table 1:** Point prevalence of hypermobility at each of the 9 sites used in the modified Beighton criteria, based on the full study population at age 13.8 years

Beighton site	Boys (n = 2,961), %	Girls (n = 3,061), %	All (n = 6,022), %
Fingers			
Left	29.9	46.6	38.4
Right	28.5	43.0	35.9
Thumbs			
Left	16.4	34.2	25.4
Right	14.0	30.0	22.1
Elbows			
Left	4.8	13.1	9.0
Right	4.4	12.4	8.5
Knees			
Left	7.8	11.2	9.6
Right	7.1	11.0	9.1
Trunk	1.7	15.1	8.5

**Table 2:** Proportion of children with hypermobility as defined using a cutoff of ≥4 hypermobile joints who were hypermobile at the individual sites, at the fingers and thumbs, or at the fingers, thumbs, and elbows

	Boys (n = 314), %	Girls (n = 842), %	All (n = 1,156), %
Fingers	84.7	85.4	85.2
Thumbs	75.2	74.7	74.8
Elbows	20.7	31.0	28.2
Knees	28.7	28.6	28.6
Trunk	4.1	25.8	19.9
Hands (fingers and thumbs)	66.6	65.9	66.1
Upper limbs (fingers, thumbs, and elbows)	4.5	12.5	10.3

The basic descriptive characteristics and potential confounding variables of generalized joint laxity in this cohort are shown in Table 3. There was no age difference between those with and those without generalized joint laxity (results not shown). Because there was evidence of an interaction between BMI and sex (*P* < 0.001), associations were assessed separately for boys and girls. None of the variables assessed showed any association with generalized joint laxity in boys (Table 4). In girls (Table 5), there was a positive association between BMI and presence of generalized joint laxity defined using a cutoff of ≥4, both without adjustment and after adjustment for all other variables (handedness, puberty, physical activity, ethnicity, and maternal education): girls who were obese were 2.7 times more likely to be hypermobile (adjusted OR 2.70 [95% CI 1.24–5.88]) compared to girls who were underweight. There was a suggestion of a similar direction of association when generalized joint laxity was defined using a cutoff of ≥6, but only from underweight through normal weight to overweight; obesity was not associated with hypermobility of ≥6 joints (although this analysis was based on only 69 girls). No other associations were seen using a cutoff of ≥4 to define generalized joint laxity.

**Table 3:** Basic descriptive characteristics of the children with and those without generalized joint laxity as defined using cutoffs of ≥4 or ≥6 hypermobile joints*

	Beighton score ≥4			Beighton score ≥6		
		
	No, no. (%)	Yes, no. (%)	*P*	No, no. (%)	Yes, no. (%)	*P*
Boys						
Handedness (n = 2,961)			0.74			0.25
Left	372 (89.9)	42 (10.1)		411 (99.3)	3 (0.7)	
Right	2,275 (89.3)	272 (10.7)		2,511 (98.6)	36 (1.4)	
BMI (n = 2,961)			0.53			0.96
Underweight	1,038 (89.9)	116 (10.1)		1,141 (98.9)	13 (1.1)	
Ideal	1,363 (88.2)	82 (11.8)		1,520 (98.4)	25 (1.6)	
Overweight	206 (94.1)	13 (5.9)		219 (100.0)	0 (0)	
Obese	40 (93.0)	3 (7.0)		42 (97.7)	1 (2.3)	
Tanner stage (n = 1,855)			0.31			0.96
I (prepubertal)	207 (90.4)	22 (9.6)		226 (98.7)	3 (1.3)	
II	389 (91.3)	37 (8.7)		420 (98.6)	6 (1.4)	
III	460 (89.7)	53 (10.3)		508 (99.0)	5 (1.0)	
IV	522 (89.1)	64 (10.9)		577 (98.5)	9 (1.5)	
V (postpubertal)	90 (89.1)	11 (10.9)		100 (99.0)	1 (1.0)	
Physical activity (n = 1,944)			0.67			0.63
<60 minutes mod/vig	1,645 (89.4)	196 (10.6)		1,814 (92.4)	27 (7.6)	
>60 minutes mod/vig	135 (88.2)	18 (11.8)		150 (90.0)	3 (10.0)	
Ethnicity (n = 2,699)			0.5			0.2
White	2,323 (89.5)	273 (10.5)		2,560 (98.6)	36 (1.4)	
Nonwhite	90 (87.4)	13 (12.6)		100 (97.1)	3 (2.9)	
Maternal education (n = 2,747)			0.37			0.92
1 (low)	302 (91.2)	29 (8.8)		328 (99.1)	3 (0.9)	
2	213 (89.1)	26 (10.9)		233 (97.5)	6 (2.5)	
3	861 (88.4)	113 (11.6)		957 (98.3)	17 (1.7)	
4	680 (90.9)	68 (9.1)		744 (99.5)	4 (0.5)	
5 (high)	403 (87.6)	57 (12.4)		451 (98.0)	9 (2.0)	
Girls						
Handedness (n = 3,061)			0.91			0.76
Left	227 (72.8)	85 (27.2)		289 (92.6)	23 (7.4)	
Right	1,992 (72.5)	757 (27.5)		2,559 (93.1)	190 (6.9)	
BMI (n = 3,061)			0.01			0.87
Underweight	649 (74.6)	221 (25.4)		808 (92.9)	62 (7.1)	
Ideal	1,335 (72.5)	507 (27.5)		1,716 (92.7)	126 (7.3)	
Overweight	192 (68.6)	88 (31.4)		259 (92.5)	21 (7.5)	
Obese	43 (62.3)	26 (37.7)		65 (94.2)	4 (5.8)	
Tanner stage (n = 1,855)			0.34			0.55
I (prepubertal)	80 (72.7)	30 (27.3)		103 (93.6)	7 (6.4)	
II	183 (74.7)	62 (25.3)		224 (91.4)	21 (8.6)	
III	372 (76.2)	116 (23.8)		461 (94.5)	27 (5.5)	
IV	605 (70.5)	253 (29.5)		788 (91.8)	70 (8.2)	
V (postpubertal)	339 (73.4)	123 (26.6)		438 (94.8)	24 (5.2)	
Physical activity (n = 2,257)			0.44			0.02
<60 minutes mod/vig	1,625 (73.4)	588 (26.6)		2,066 (93.4)	147 (6.6)	
>60 minutes mod/vig	30 (68.2)	14 (31.8)		37 (84.1)	7 (15.9)	
Ethnicity (n = 2,782)			0.33			0.24
White	1,937 (72.3)	742 (27.7)		2,495 (93.1)	184 (6.9)	
Nonwhite	79 (76.7)	24 (23.3)		99 (96.1)	4 (3.9)	
Maternal education (n = 2,812)			0.63			0.01
1 (low)	259 (73.2)	95 (26.8)		343 (96.9)	11 (3.1)	
2	164 (77.7)	47 (22.3)		203 (96.2)	8 (3.8)	
3	694 (70.5)	291 (29.5)		906 (92.0)	79 (8.0)	
4	571 (73.4)	207 (26.6)		725 (93.2)	53 (6.8)	
5 (high)	355 (72.0)	138 (28.0)		453 (91.9)	40 (8.1)	

* BMI = body mass index; mod/vig = moderate and/or vigorous daily physical activity.

**Table 4:** Odds ratios for the presence of generalized joint laxity (as defined using cutoffs of ≥4 or ≥6 hypermobile joints) in boys, according to variables of interest*

	Beighton score ≥4		Beighton score ≥6	
		
	Unadjusted OR (95% CI)	Adjusted OR (95% CI)†	Unadjusted OR (95% CI)	Adjusted OR (95% CI)†
Handedness				
Left	1.0 (referent)	1.0 (referent)	1.0 (referent)	1.0 (referent)
Right	1.06 (0.75–1.49)	1.22 (0.69–2.16)	1.96 (0.60–6.41)	3.17 (0.42–24.06)
BMI				
Underweight	1.0 (referent)	1.0 (referent)	1.0 (referent)	1.0 (referent)
Ideal	1.20 (0.93–1.53)	1.31 (0.88–1.95)	1.44 (0.74–2.83)	1.87 (0.70–5.04)
Overweight	0.57 (0.31–1.02)	0.36 (0.11–1.18)	NA	NA
Obese	0.67 (0.20–2.20) (OR test for trend 0.95 [95% CI 0.79–1.13])	1.57 (0.18–13.33) (OR test for trend 0.97 [95% CI 0.72–1.32])	2.09 (0.27–16.4) (OR test for trend 1.01 [95% CI 0.63–1.63])	NA (OR test for trend 1.07 [95% CI 0.52–2.18])
Tanner stage				
I (prepubertal)	1.0 (referent)	1.0 (referent)	1.0 (referent)	1.0 (referent)
II	0.90 (0.51–1.56)	0.94 (0.49–1.79)	1.08 (0.27–4.34)	0.92 (0.21–3.94)
III	1.08 (0.64–1.83)	0.73 (0.39–1.40)	0.74 (0.18–3.13)	0.51 (0.11–2.32)
IV	1.15 (0.69–1.92)	0.85 (0.46–1.58)	1.18 (0.32–4.38)	0.79 (0.20–3.20)
V (postpubertal)	1.15 (0.54–2.47) (OR test for trend 1.07 [95% CI 0.94–1.23])	0.76 (0.28–2.07) (OR test for trend 0.96 [95% CI 0.81–1.14])	0.75 (0.08–7.33) (OR test for trend 1.01 [95% CI 0.70–1.45])	0.69 (0.07–7.05) (OR test for trend 0.94 [95% CI 0.63–1.41])
Physical activity				
<60 minutes mod/vig	1.0 (referent)	1.0 (referent)	1.0 (referent)	1.0 (referent)
>60 minutes mod/vig	1.12 (0.67–1.87)	1.73 (0.93–3.19)	1.34 (0.40–4.48)	1.48 (0.33–6.59)
Ethnicity				
White	1.0 (referent)	1.0 (referent)	1.0 (referent)	1.0 (referent)
Nonwhite	1.23 (0.68–2.23)	0.40 (0.05–2.93)	2.13 (0.64–7.05)	NA
Maternal education				
1 (low)	1.0 (referent)	1.0 (referent)	1.0 (referent)	1.0
2	1.25 (0.72–2.18)	0.97 (0.37–2.52)	2.77 (0.69–11.2)	NA
3	1.34 (0.88–2.06)	1.21 (0.58–2.51)	1.91 (0.56–6.57)	NA
4	1.02 (0.65–1.62)	0.73 (0.33–1.58)	0.58 (0.13–2.60)	NA
5 (high)	1.45 (0.90–2.32) (OR test for trend 1.04 [95% CI 0.94–1.15])	1.31 (0.61–2.82) (OR test for trend 1.02 [95% CI 0.87–1.20])	2.15 (0.58–8.00) (OR test for trend 0.98 [95% CI 0.75–1.28])	NA (OR test for trend 1.30 [95% CI 0.86–1.98])

* No signficant associations with any of the variables were identified. OR = odds ratio; 95% CI = 95% confidence interval; NA = not available (analysis could not be performed because of small numbers) (see Table 3 for other definitions).

† Adjusted for all other variables shown.

**Table 5:** Odds ratios for the presence of generalized joint laxity (as defined using cutoffs of ≥4 or ≥6 hypermobile joints) in girls, according to variables of interest*

	Beighton score ≥4		Beighton score ≥6	
		
	Unadjusted OR (95% CI)	Adjusted OR (95% CI)†	Unadjusted OR (95% CI)	Adjusted OR (95% CI)†
Handedness				
Left	1.0 (referent)	1.0 (referent)	1.0 (referent)	1.0 (referent)
Right	2 (0.78–1.32)	0.94 (0.64–1.40)	0.93 (0.60–1.46)	0.77 (0.41–1.46)
BMI				
Underweight	1.0 (referent)	1.0 (referent)	1.0 (referent)	1.0 (referent)
Ideal	1.12 (0.93–1.34)	1.36 (1.03–1.80)	0.96 (0.70–1.32)	1.38 (0.85–2.25)
Overweight	1.35 (1.01–1.81)	2.13 (1.37–3.30)	1.06 (0.63–1.77)	1.74 (0.81–3.73)
Obese	1.78 (1.07–2.96) (OR test for trend 1.17 [95% CI 1.04–1.32], *P* = 0.009)	2.70 (1.24–5.88) (OR test for trend 1.39 [95% CI 1.17–1.67], *P* < 0.001)	0.80 (0.28–2.27) (OR test for trend 0.98 [95% CI 0.80–1.21])	0.81 (0.10–6.37) (OR test for trend 1.18 [95% CI 0.88–1.63])
Tanner stage				
I (prepubertal)	1.0 (referent)	1.0 (referent)	1.0 (referent)	1.0 (referent)
II	0.90 (0.54–1.51)	1.05 (0.55–1.99)	1.38 (0.57–3.35)	1.90 (0.52–6.89)
III	0.83 (0.52–1.33)	1.08 (0.60–1.95)	0.86 (0.37–2.03)	1.52 (0.44–5.24)
IV	1.12 (0.72–1.74)	1.20 (0.68–2.12)	1.31 (0.59–2.92)	1.90 (0.57–6.34)
V (postpubertal)	0.97 (0.61–1.54) (OR test for trend 1.04 [95% CI 0.96–1.14])	0.93 (0.51–1.70) (OR test for trend 0.99 [95% CI 0.89–1.11])	0.81 (0.34–1.92) (OR test for trend 0.96 [95% CI 0.82–1.11])	1.09 (0.30–3.93) (OR test for trend 0.97 [95% CI 0.81–1.18])
Physical activity				
<60 minutes mod/vig	1.0 (referent)	1.0 (referent)	1.0 (referent)	1.0 (referent)
>60 minutes mod/vig	1.29 (0.68–2.45)	1.29 (0.57–2.92)	2.66 (1.17–6.07)‡	2.87 (1.04–7.91)‡
Ethnicity				
White	1.0 (referent)	1.0 (referent)	1.0 (referent)	1.0 (referent)
Nonwhite	0.79 (0.50–1.26)	0.90 (0.48–1.66)	0.55 (0.20–1.51)	0.48 (0.12–3.02)
Maternal education				
1 (low)	1.0 (referent)	1.0 (referent)	1.0 (referent)	1.0 (referent)
2	0.78 (0.52–1.16)	0.78 (0.40–1.49)	1.32 (0.51–3.40)	0.83 (0.19–3.57)
3	1.14 (0.86–1.50)	1.65 (1.05–2.57)	2.92 (1.50–5.71)	2.24 (0.86–5.84)
4	0.98 (0.74–1.31)	1.37 (0.87–2.16)	2.45 (1.23–4.87)	1.82 (0.68–4.88)
5 (high)	1.05 (0.77–1.43) (OR test for trend 1.02 [95% CI 0.95–1.09])	1.36 (0.84–2.12) (OR test for trend 1.07 [95% CI 0.97–1.18])	2.96 (1.46–6.00) (OR test for trend 1.21 [95% CI 1.06–1.38], *P* = 0.004)	3.13 (1.18–8.36) (OR test for trend 1.3 [95% CI 1.08–1.57], *P* = 0.006)

* *P* values that were significant in tests for trend are shown. OR = odds ratio; 95% CI = 95% confidence interval (see Table 3 for other definitions).

† Adjusted for all other variables shown.

‡ *P* < 0.05.

When generalized joint laxity was defined using a cutoff score of ≥6, a strong positive association between physical activity and generalized joint laxity was seen, with girls performing moderate or vigorous physical activity for >60 minutes per day being almost 3 times more likely to be hypermobile, after adjustment for all other variables (OR 2.87 [95% CI 1.04–7.91]). A trend toward a similar association was seen when generalized joint laxity was defined using a cutoff of ≥4 joints. A positive association with increasing maternal education was also seen (OR for presence of generalized joint laxity in girls whose mothers had a university degree 3.13 [95% CI 1.18–8.36] compared to girls whose mothers had no formal education). There was a trend toward a similar association in those with generalized joint laxity defined using a cutoff score of ≥4, after adjustment for all other variables.

## DISCUSSION

In this first population-based cohort study of generalized joint laxity in children from the UK, the prevalence of generalized joint laxity in girls and boys age 13.8 years was 27.5% and 10.6%, respectively, when the commonly used cutoff of ≥4 hypermobile joints from the modified Beighton 9-point scoring system was used. This provides the first population-based point prevalence data on 14-year-old children from the UK, and the data fit well with estimates previously reported in the literature (being approximately mid-range in relation to the other estimates). Girls were more likely to be hypermobile at the fingers, thumbs, and trunk, whereas boys were most often hypermobile at the fingers, thumbs, and knees. More than 40% of girls showed hyperextensibility at the little finger, leading to the conclusion that this may be normal in a teenage population. Similarly, >30% of girls scored positively for thumb apposition.

It is interesting that the lumbar spine was considerably less hypermobile, particularly in boys. This may be explained by the fact that the majority of lumbar flexion is a combination of hamstring extension and actual vertebral flexion (32), and short hamstrings have been associated with reduced lumbar flexion in men (33). It is possible that short hamstrings may have contributed to a perceived reduction in lumbar flexion, and could explain the low prevalence of lumbar hypermobility among boys in our study.

This study also provides the first reported population-based basic descriptive characteristics and information on associations with potential confounding variables in generalized joint laxity in adolescents. No associations were found in boys, possibly because of small numbers. However, among girls, generalized joint laxity was shown to be positively associated with levels of physical activity, BMI, and mother's education level. Girls who underwent >60 minutes of moderate to vigorous physical activity per day were almost 3 times as likely to have generalized joint laxity than those who were not active. We are unable to provide evidence that certain sports are associated with generalized joint laxity because our method of assessing activity was by accelerometry, which does not distinguish between activity types. Nonetheless, as children who do gymnastics or ballet, for example, are likely to be generally more active than children who do not (34), our study supports the results from previous studies showing that children who have a higher range of joint movement may be involved in certain sports or music activities (18–21).

Among girls in the present study, those whose mothers had a university degree were ∼3 times as likely to have hypermobility than those whose mothers had no formal education. This is in direct contrast to the findings in a previous study from Mumbai, India (35), in which moderate and severe malnutrition were associated with generalized joint laxity, suggesting that lower socioeconomic level may be a factor. Conversely, our study suggests that in the UK, lifestyle choices of families in which the mothers have a university degree are associated with generalized joint laxity in the children. For example, ballet dancing or gymnastics may either maintain the presence of hypermobility or perhaps promote hypermobility through forced hyperextension.

We also found an independent positive association between BMI and generalized joint laxity in girls when generalized joint laxity was defined using a cutoff of ≥4, and a suggestion of a similar association when a cutoff of ≥6 was used. This is in direct contrast to the results of previous studies in which no association between joint hypermobility and BMI was demonstrated (8, 24, 25). However, those earlier studies had much smaller study populations than the present study, and therefore had less power to assess this relationship clearly.

In our study, there was little evidence for laterality of hypermobility, consistent with the findings of one previous small study but in contrast to another (22, 23). In addition, there was no evidence of an association with ethnicity, although only a small proportion of the ALSPAC cohort is nonwhite (3.7%). Interestingly, we also showed no association with either age or puberty. This is consistent with results of the largest of the previous studies (15), but contradicts the generally held belief that generalized joint laxity lessens with aging and growth during childhood. Although the small age range among the subjects in our study might have explained the lack of association with age, we had sufficient numbers of children in each stage of puberty, and thus any relationship between maturational status and joint hypermobility would have been evident had one been present. A further limitation of our study includes loss of a large proportion of the original ALSPAC cohort, which may have introduced bias, for example, by a preferential dropout of children from families of lower socioeconomic status. In common with all observational studies, we cannot exclude confounding and chance, and in this report we describe associations but are not attempting to comment on temporal relationships or causality.

In conclusion, using the standard cutoff of ≥4 hypermobile joints, 1,156 of the 6,022 school-age children in the present study would currently receive a diagnosis of generalized joint laxity. This suggests that a Beighton score of 4 is too low a cutoff for use in identifying children with a pathologic entity. Increasing the threshold for diagnosing this condition, for example by raising the Beighton score cutoff, should result in a smaller proportion of children being diagnosed, in whom risk factors and pathologic sequelae may be easier to detect. We found stronger evidence of associations with physical activity and maternal education when generalized joint laxity was defined based on a Beighton score cutoff of ≥6 compared with ≥4. A reasonable alternative to raising the Beighton score cutoff for diagnosis of generalized joint laxity might be to exclude digits from the definition, since hypermobility of the little finger is essentially normal given its presence in >40% of girls. Finally, there may be a need to devise a new, more specific assessment tool to evaluate joint laxity in the developing musculoskeletal system—one that can be used to identify children at risk of symptoms such as pain and pathology such as connective tissue disease and, as importantly, to reassure those who do not need further medical intervention.
